# Association between 6:2 chlorinated polyfluoroalkyl ether sulfonic acid exposure and glucolipid metabolism in Chinese adults: a meta-analysis

**DOI:** 10.1186/s13643-026-03067-3

**Published:** 2026-01-16

**Authors:** Qing Chen, Tao Ying, Hua Cai, Hong Liu, Geng-sheng He

**Affiliations:** 1 School of Public Health, Key Laboratory of Public Health Safety of the Ministry of Education, Fudan University, 130 Dong’an Road, Shanghai, 200032 China; 2 Shanghai Municipal Center for Disease Control and Prevention, Shanghai, 200336 China

**Keywords:** Data synthesis, Human exposure, Glucolipid metabolism, 6:2 Cl-PFESA

## Abstract

**Background:**

In China, 6:2 chlorinated polyfluoroalkyl ether sulfonic acid (6:2 Cl-PFESA), commercially designated as F-53B, has been predominantly implemented as the perfluorooctane sulfonate (PFOS) substitute, with its 8:2 derivative showing negligible relevance to population exposure. Previous studies have found that 6:2 Cl-PFESA is closely related to dyslipidemia and disrupted glucose homeostasis, though results remain inconsistent.

**Methods:**

Our study aimed to separately investigate the potential associations of 6:2 and 8:2 Cl-PFESA with glucolipid metabolism indicators in Chinese adults. A comprehensive systematic literature search was conducted across three major databases—PubMed, Embase, and Web of Science—by 23 September 2024. Random effects models were employed to estimate changes in blood lipid and glucose parameters with one interquartile range (IQR) increment in mean blood concentration of 6:2 and 8:2 Cl-PFESA. Subgroup analyses were performed based on different populations and types of diabetes. A Bayesian random-effects meta-regression model was conducted to attribute differences apparent between individual empirical estimates to mean 6:2 Cl-PFESA concentration.

**Results:**

The analysis included 17 publications with more than 17, 000 participants. Meta-analyses revealed that each IQR increase of 6:2 Cl-PFESA exhibited significantly positive correlations with a 3.90 mg/dl change in total cholesterol (TC) (95% CI: 1.97, 5.83), and a 2.94 mg/dl change in low-density lipoprotein cholesterol (LDL-C) (95% CI: 1.47, 4.41) among the general adult population. The odds ratio (OR) for gestational diabetes mellitus (GDM) among pregnant women exposed to 6:2 Cl-PFESA was 1.61 (95% CI: 1.15, 2.27). Studies on 8:2 Cl-PFESA were insufficient.

**Conclusion:**

The results demonstrated that 6:2 Cl-PFESA was significantly related to TC, LDL-C, as well as the risk of GDM. These findings challenge the current industrial designation of 6:2 Cl-PFESA as a safer alternative, necessitating explicit regulatory re-evaluation pending comprehensive mechanistic evidence that elucidates its biological interactions.

**Systematic review registration:**

PROSPERO CRD42024581843

**Supplementary Information:**

The online version contains supplementary material available at 10.1186/s13643-026-03067-3.

## Introduction

F-53B, primarily composed of 6:2 chlorinated polyfluoroalkyl ether sulfonic acid (6:2 Cl-PFESA) with minor amounts of 8:2 Cl-PFESA, has been adopted for the preparation of chromium mist suppressants in the Chinese electroplating sector, strategically substituting perfluorooctane sulfonate (PFOS)—the prototypical type of legacy per- and polyfluoroalkyl substances (PFAS). 6:2 Cl-PFESA exhibits structural analogy to PFOS, retaining the hydrophobic perfluorinated chain and hydrophilic sulfonate group essential for surface activity. With the introduction of a chlorine atom and an ether linkage to simplify synthesis and lower costs, these modifications have prompted assertions of improved safety profiles, including reduced environmental persistence and toxicity relative to PFOS [1]. Nevertheless, the conserved structural features imply that 6:2 Cl-PFESA may exert detrimental effects on glucolipid metabolism analogous to those of PFOS. This discrepancy underscores the necessity for rigorous validation of these purported safety benefits.

No clear consensus has been reached on the relationship between 6:2 Cl-PFESA and disruptions in glucolipid metabolism. A significant decline in triglycerides (TG) and total cholesterol (TC) was observed in adult male mice following 6:2 Cl-PFESA treatment [2], while a considerable increase in serum glucose content was found in mature zebrafish subjected to 6:2 Cl-PFESA exposure [3]. Meanwhile, epidemiological efforts have been undertaken to assess associations of 6:2 Cl-PFESA with glucolipid metabolic markers, including diabetes, blood glucose, and lipid profiles [4, 5]. Furthermore, emerging evidence suggests that 6:2 Cl-PFESA may interfere with key metabolic regulators such as peroxisome proliferator-activated receptors (PPARs) and glucokinase, potentially disrupting glucose and lipid homeostasis [6, 7].

Currently, the internal exposure concentration of 6:2 Cl-PFESA is among the top three highest PFAS in the Chinese population [8]. Distinct exposure gradients of 6:2 Cl-PFESA were observed across different population groups, reaching maximum levels of 5.31 ng/ml in maternal-neonatal dyads, 9.12 ng/ml in the general population, and 941 ng/ml in occupationally exposed electroplating workers [9]. Moreover, the estimated weekly intakes of 6:2 Cl-PFESA, as derived by the Sixth China Total Diet Study (TDS), have reached 51.57 ng/kg·bw/week [10], almost four times the tolerable weekly intake of PFOS established by the European Food Safety Authority (EFSA) (13 ng/kg·bw/week) [11]. Coincidentally, 6:2 Cl-PFESA shows a global distribution, with detections reported in Korea, Europe, and the USA [12]. This widespread occurrence and potential for significant exposure necessitate increased vigilance regarding the health risks of 6:2 Cl-PFESA.

As no previous meta-analysis has been undertaken, we conducted this first meta-analysis to synthesize the emerging human epidemiological evidence on the association of exposure to 6:2 and 8:2 Cl-PFESA with glucolipid metabolism indicators in Chinese adults, including 4 lipid profiles—TG, TC, low-density lipoprotein cholesterol (LDL-C), high-density lipoprotein cholesterol (HDL-C), and 4 glucose homeostasis parameters—fasting blood glucose (FBG), 1 h and 2 h glucose levels, and diabetes. Given the widespread exposure and inconsistent metabolic evidence of 6:2 Cl-PFESA, we therefore performed subgroup analyses and meta-regressions to elucidate the underlying sources of heterogeneity. Log transformations for exposure measures (6:2 and 8:2 Cl-PFESA) and continuous variables in different literatures were also considered in our analyses. The scarcity of epidemiological data on 6:2 and 8:2 Cl-PFESA beyond China has limited this meta-analysis to studies of Chinese populations.

## Methods

### Search strategy

We searched PubMed, Embase, and Web of Science for studies published before 23 September 2024. The search was conducted using the following terms:

- #1 Cl-PFAES OR Cl-PFESA OR F-53B OR F53B OR PFESA OR PFAES OR (chlorinated AND polyfluor* AND ether AND sulfon*) OR ((novel OR emerging OR alternati* OR new OR generation) AND (perfluor* OR polyfluor* OR PFAS* OR PFOS OR perfluorooctane* OR PFOA))
- #2 glucose OR “Glu” OR “blood sugar” OR “FPG” OR “FBG” OR “FBS” OR glycemic OR “glycosylated hemoglobin” OR “glycated hemoglobin” OR “GHB” OR “HbA1c” OR “HBALC” OR insulin OR hyperinsulinemia OR “HOMA IR” OR INS OR FINS OR IRI OR diabetes OR GDM OR “gestational diabetes mellitus” OR T2DM
- #3 lipid OR fat OR “LDL” OR “low density lipoprotein” OR cholesterol OR cholesterin OR cholestenone OR “CHOL” OR “TC” OR “HDL” OR “high density lipoprotein” OR triglyceride OR triacylglycerol OR “TG” OR “TAG” OR “TRIG”

#### #1 AND (#2 OR #3)

**Fig. 1:**
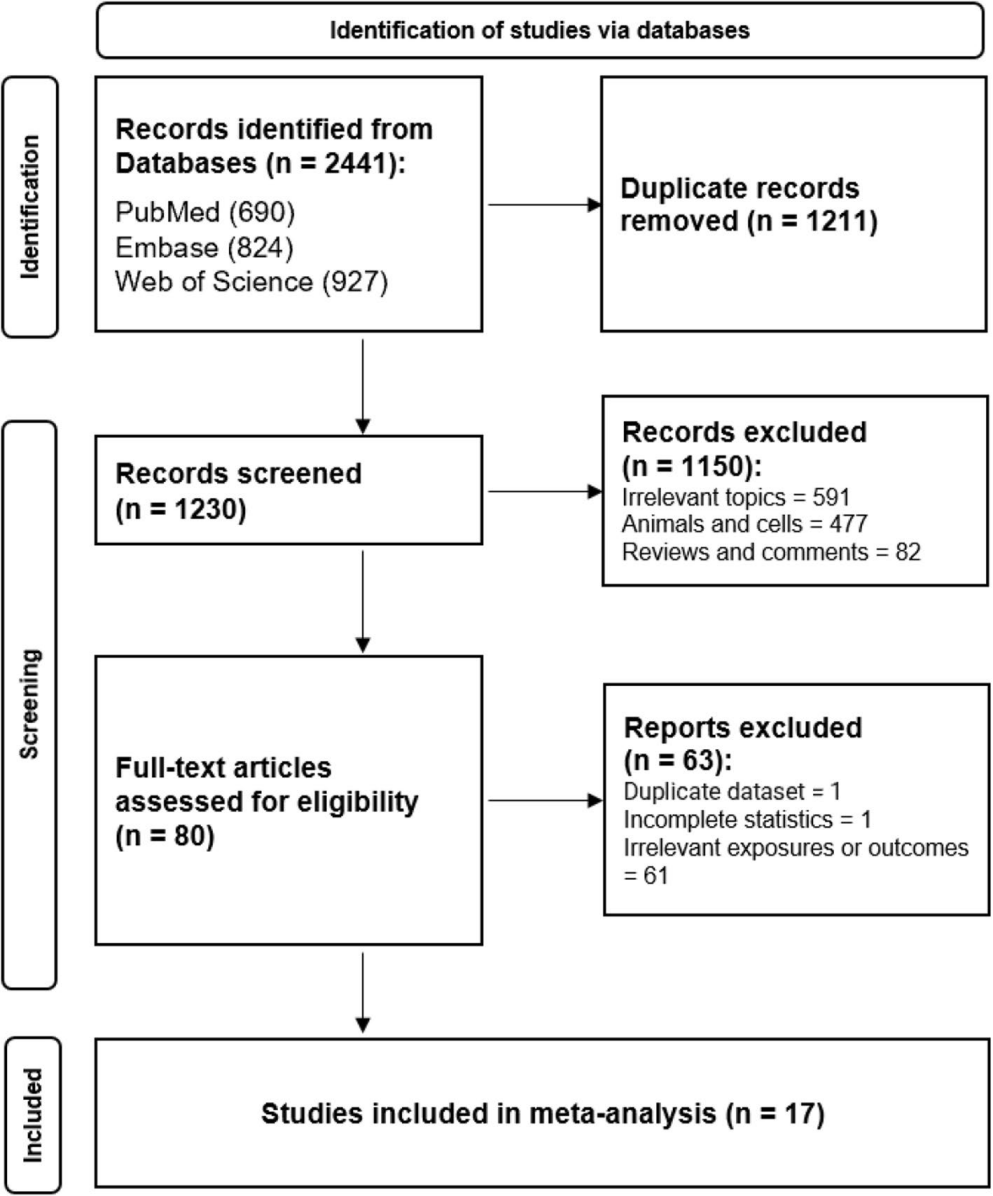
Flow diagram of retrieved eligible articles

Two investigators (Q.C., T.Y.) independently conducted the screening of articles, followed by the flow diagram (Fig. 1). Discrepancies were resolved via consultation with the senior author. References of included studies were reviewed manually as well. All retrieved articles were imported into EndNote 20 for reference management. A predefined protocol was recorded for the meta-analysis (PROSPERO registration number: CRD42024581843). The protocol was developed following the Preferred Reporting Items for Systematic review and Meta-Analysis Protocols (PRISMA-P) guidelines (Supplementary Appendix A).

#### Selection criteria

Inclusion criteria are listed below. Studies centered on associations of exposure to 6:2 and 8:2 Cl-PFESA with glucolipid metabolism were included. Population-based epidemiological studies (e.g., cohort, case–control, or cross-sectional designs) were included. Studies that reported valid and complete data were included. Exclusion criteria are as follows. Studies with irrelevant research topics or outcomes were excluded. Studies on animals, cells, and other non-population-based studies were excluded. Reviews, conference reports, abstracts, editorials, and letters were excluded. Studies with duplicate datasets or overlapping populations were excluded. Studies lacking essential statistical information (e.g., effect estimates, measures of variation, or sample sizes) were excluded.

### Data extraction

The detailed information of eligible studies was independently extracted by Q.C. and T.Y. as follows: literature citation, study design and participant recruitment, sample size, average age, sex, 6:2 and 8:2 Cl-PFESA exposure, covariate adjustment, and effect estimates [β coefficients, odds ratio (OR), and 95% confidence intervals (CI)]. Notably, the web-based tool WebPlotDigitizer was used to extract the numerical data from graphs in two included studies by Han and Hu [13, 14].

### Quality evaluation

The Newcastle–Ottawa Scale (NOS) was employed to assess the quality. The score of NOS was classified into three levels, of which 7–9 represents high, 4–6 represents moderate, and below 3 represents low (Table-S1 and S-2) [15]. The scale consists of eight items across three areas, where case–control studies assess exposure and cohort studies assess outcomes. 6:2 Cl-PFESA exhibits a prolonged biological half-life of 15.3 years [16]. Given its high persistence, a single serum measurement likely reflects chronic exposure burden, thereby reducing exposure misclassification that often complicates epidemiological studies of non-persistent compounds. Moreover, the reporting quality of the meta-analysis was evaluated using PRISMA 2020, whereas its methodological quality was assessed with A Measurement Tool to Assess Systematic Reviews (AMSTAR) 2 (Supplementary Appendix B-C). The overall certainty of evidence for the primary outcomes was assessed using the Grade of Recommendations Assessment, Development and Evaluation (GRADE) approach (Table S6). Additional details can be found in the supplementary materials.

### Statistical analysis

This meta-analysis evaluated the effect estimates for differences in blood lipids or glucose levels per interquartile range (IQR) increase in blood 6:2 and 8:2 Cl-PFESA exposure, following the methodology originated from the preceding PFAS-related systematic review and meta-analysis [17]. Using the IQR for analysis not only captures the variability of 6:2 and 8:2 Cl-PFESA exposure, which typically follows a skewed distribution, but also ensures comparability across heterogeneous datasets. The log transformation of 6:2 and 8:2 Cl-PFESA exposure and metabolism indexes (*Y*) for glucose and lipids varies across different studies. Accordingly, for models with log-transformed variables, the corresponding regression coefficients were interpreted as representing percentage changes rather than absolute changes. For linear–linear transformation, changes of *Y* (mg/dl) per IQR increase in 6:2 and 8:2 Cl-PFESA (ng/ml) were calculated as Δ = *β* × *IQR* (*β* was the reported regression coefficient, *IQR* was the IQR of 6:2 and 8:2 Cl-PFESA level). For linear-log transformation, Δ = *β* × *IQR*_log_ [*IQR*_log_ was the IQR of log(6:2 and 8:2 Cl-PFESA)]. For log-linear transformation, Δ = $\overline{Y }$×*β* × *IQR* ($\overline{Y }$ is the mean of raw data). For log–log transformation, Δ = $\overline{Y }$×*β* × *IQR*_log_. Further details are provided in the Supplementary Information. The summary OR for diabetes was determined by comparing the highest and lowest concentrations of categorical 6:2 and 8:2 Cl-PFESA exposure. Random effects models were performed to calculate the combined effect size (ES) and standard error (SE).

Heterogeneity was evaluated by the Cochran Q and I^2^ statistics. To investigate potential sources of heterogeneity, we conducted subgroup analyses and univariable meta-regressions. Subgroup analyses were stratified by different populations and types of diabetes. Univariable meta-regression was performed using the following covariates: age, sex, study design, sample size, population characteristics, 6:2 and 8:2 Cl-PFESA exposure concentration, and the number of covariates adjusted for in the original studies (Table S8). Stability of selected studies was determined according to sensitivity analyses, encompassing both leave-one-out approach and targeted exclusion of outlier studies. Publication bias was appraised by Egger’s regression test and Begg’s rank test. For associations with evidence of publication bias, ​​we performed​​ trim-and-fill analysis to impute missing studies and compute ​​an​​ adjusted effect estimate.

To quantitatively assess the dose–response relationship between 6:2 Cl-PFESA exposure and glucolipid metabolism while controlling for potential demographic confounding, we performed a Bayesian meta-regression model. In this model, the pooled effect size was specified as the outcome variable, with the mean 6:2 Cl-PFESA concentration as the primary continuous predictor. The model was further adjusted for age and sex as covariates. The Bayesian framework offers the advantage of incorporating prior knowledge and providing full posterior distributions for each association, which is particularly advantageous in the presence of limited studies or high heterogeneity. The statistical analyses were carried out using R packages (“metafor” and “bayesmeta” in version 4.4.2). The mean of blood 6:2 and 8:2 Cl-PFESA was represented by the median value extracted from the included studies. The statistical significance was established for *P* values below than 0.05 (two-sided).

## Results

### Study inclusion

A total of 2441 articles were primarily retrieved from three electronic databases, and 1230 were retained after removal of 1211 duplicates. Excluded studies contained 82 non-original works and 591 unrelated to the link between 6:2 and 8:2 Cl-PFESA exposure and glucolipid metabolism. Studies on animals, cells, and other non-population-based studies (*n* = 477) were excluded as well. During the data extraction stage of full-text assessment, studies with incomplete statistics (*n* = 1) [18], duplicate datasets for the same outcomes (*n* = 1) [4], and irrelevant exposures or outcomes (*n* = 61) were excluded. Ultimately, 17 studies were incorporated into our meta-analysis (Fig. 1).

### Study summary

Table 1 lists the basic information of 17 included studies, all of which were published in the last 5 years and conducted from all over China, including Beijing, Shanghai, Guangdong, Zhejiang, Tianjin, Hubei, Shandong, Sichuan, and Liaoning. The incorporated studies primarily utilized observational epidemiological designs, including cross-sectional (*n* = 9), case–control (*n* = 3), cohort (*n* = 3), and nested case–control studies (*n* = 3), with sampling years ranging from 2011 to 2022. More than seventeen thousand participants were included. All studies contained the results of 6:2 Cl-PFESA exposure with measured mean concentration ranging from 0.32 to 9.36 ng/ml, and only 9 studies mentioned results of 8:2 Cl-PFESA with mean from 0.01 to 2.42 ng/ml. For the general adult population, eight studies reported results of serum lipids, and two studies reported diabetes-related conditions, including type 2 diabetes mellitus (T2DM) and prediabetes. For pregnant women, four studies presented blood glucose levels and six studies reported risks of gestational diabetes mellitus (GDM). NOS scores ranged from 5 to 9.

**Table 1:** Basic information of included studies (*N* = 17)

Compounds	Outcome	Study design	Location	Sample size	Percentage of males (%)	Age (years)	Participant recruitment	Recruitment time	Variables adjusted	Reference
6:2 Cl-PFESA	Prediabetes, Diabetes, FBG	cross-sectional	China	10,851	61.2	46.7	China National Human Biomonitoring (CNHBM) survey	2017–2018	Age, sex, residence, smoking, drinking, activity time, family history of diabetes, food consumption	Qu et al., 2024 [5]
6:2 Cl-PFESA	HDL-C, LDL-C, TC, TG	cross-sectional	Jinan, Shandong, China	575	64.9	43.3	Annual medical examinations at a hospital in Jinan City, Shandong Province, China	2022	Age, sex, BMI, occupation, education	Liu et al., 2024 [19]
6:2 Cl-PFESA	HDL-C, LDL-C, TC, TG	cross-sectional	China	10,855	61.1	44.7	CNHBM	2017–2018	Age, sex, BMI, education, marital status, residence, nationality, household income, smoking, drinking, food consumption, hypertension, eGFR, diabetes	Wu et al., 2023 [20]
6:2 Cl-PFESA	HDL-C, LDL-C, TC, TG	cross-sectional	Laizhou Bay, Shandong, China	161	66.0	34.0	Residents living in Laizhou Bay, Shandong, China	2017	Age, sex, BMI, education, smoking	Liu et al., 2023b [21]
6:2 Cl-PFESA	FBG	cross-sectional	Shenyang, Liaoning, China	923	51.1	62.0	Isomers of C8 Health Project in China	2015–2016	Age, sex, BMI, nationality, household income, education, occupation, smoking, drinking, exercise, aquatic product intake	Huang et al., 2023a [22]
6:2 Cl-PFESA 8:2 Cl-PFESA	HDL-C, LDL-C, TC, TG	cross-sectional	Guangzhou, Guangdong, China	1,336	40.1	53.4	Residents living in Guangzhou, China	2018–2019	Age, sex, household income, education, marital status, smoking, exercise	Mi et al., 2022 [23]
6:2 Cl-PFESA	HDL-C, LDL-C, TC, TG, FBG, T2DM	case–control	Shandong, China	153	34.0	50.0	Shandong Provincial Qianfoshan Hospital, Shandong Province, China	2016–2017	Age, sex, BMI	Han et al., 2021 [13]
6:2 Cl-PFESA 8:2 Cl-PFESA	HDL-C, LDL-C, TC, TG	cross-sectional	Shenyang, Liaoning, China	1238	54.9	62.0	Isomers of C8 Health Project in China	2015–2016	Age, sex, ethnicity, education, household income, occupation, smoking, drinking, exercise	Cong et al., 2021 [24]
6:2 Cl-PFESA	HDL-C, LDL-C, TC, TG	cross-sectional	Huantai County, Shandong, China	311	50.8	48.0	Residents at the People’s Hospital of Huantai County, near fluoropolymer plant in Huantai County, Shandong, China	2019–2020	Age and sex	Yao et al., 2020 [25]
6:2 Cl-PFESA 8:2 Cl-PFESA	FBG	cross-sectional	Tianjin, China	252	37.7	51.0	Staff and support workers in Nankai University, Tianjin, China	2017	Age, sex, BMI, smoking, drinking, exercise, education, history of diabetes	Duan et al., 2020 [26]
6:2 Cl-PFESA 8:2 Cl-PFESA	FBG, 1-h, and 2-h glucose levels, GDM	cohort	Shanghai, China	336	0.0	33.0	International Peace Maternity & Child Health Hospital, Shanghai, China	2017–2019	Age, pre-pregnancy BMI, education, smoking, parity, fish intake	Mao et al., 2024 [27]
6:2 Cl-PFESA	GDM	case–control	Hangzhou, Zhejiang, China	204	0.0	33.2	Women’s Hospital School of Medicine Zhejiang University, China	2011–2012	Age, maternal BMI, fetal sex, parity	Zhang et al., 2023 [28]
6:2 Cl-PFESA 8:2 Cl-PFESA	FBG, 1-h, and 2-h glucose levels, GDM	nested case–control	Shanghai, China	295	0.0	32.0	International Peace Maternity & Child Health Hospital, Shanghai, China	2019–2020	Age, pre-pregnancy BMI, education, parity, spontaneous abortion times, pregnancy mode, sampling time, TG, TC	Zang et al., 2023 [29]
6:2 Cl-PFESA 8:2 Cl-PFESA	GDM	nested case–control	Chengdu, Sichuan, China	360	0.0	27.8	Tongji-Shuangliu Birth Cohort (TSBC)	2017–2019	Age, gestational age, pre-pregnancy BMI, education, smoking, drinking, history of GDM, parental diabetes, exercise	Huang et al., 2023b [30]
6:2 Cl-PFESA	HDL-C, LDL-C, TC, TG	cohort	Beijing, China	118	0.0	33.6	Nutrition clinic of Haidian District Maternal & Child Health Hospital, Beijing, China	2017	Age, pre-pregnancy BMI, parity, gestational age of enrollment, income, macrosomia history	Hu et al., 2023 [14]
6:2 Cl-PFESA 8:2 Cl-PFESA	FBG, 1-h, and 2-h glucose levels, GDM	case–control	Hangzhou, Zhejiang, China	340	0.0	31.5	Women’s Hospital School of Medicine Zhejiang University, China	2020–2021	Age, pre-pregnancy BMI, education, occupation, smoking, drinking, ethnicity, menstrual cycle, menarche age, parity	Xu et al., 2022 [31]
6:2 Cl-PFESA 8:2 Cl-PFESA	FBG, 1-h, and 2-h glucose levels, GDM	cohort	Wuhan, Hubei, China	874	0.0	28.0	Women and Children Medical and Healthcare Center of Wuhan, Hubei, China	2013–2014	Age at pregnancy, pre-pregnancy BMI, education, household income, passive smoking during pregnancy, infant sex, parity	Li et al., 2020 [32]

The population characteristic was dichotomized based on the variable "percentage of males (%)", where "0" indicated pregnant women and any other value represented the general population

*FBG* fasting blood glucose, *HDL-C* high-density lipoprotein cholesterol, *LDL-C* low-density lipoprotein cholesterol, *TC* total cholesterol, *TG* triacylglycerol, *GDM* gestational diabetes mellitus, *T2DM* type 2 diabetes mellitus, *BMI* body mass index

### 6:2 Cl-PFESA, glucolipid metabolism, and subgroup results

**Fig. 2:**
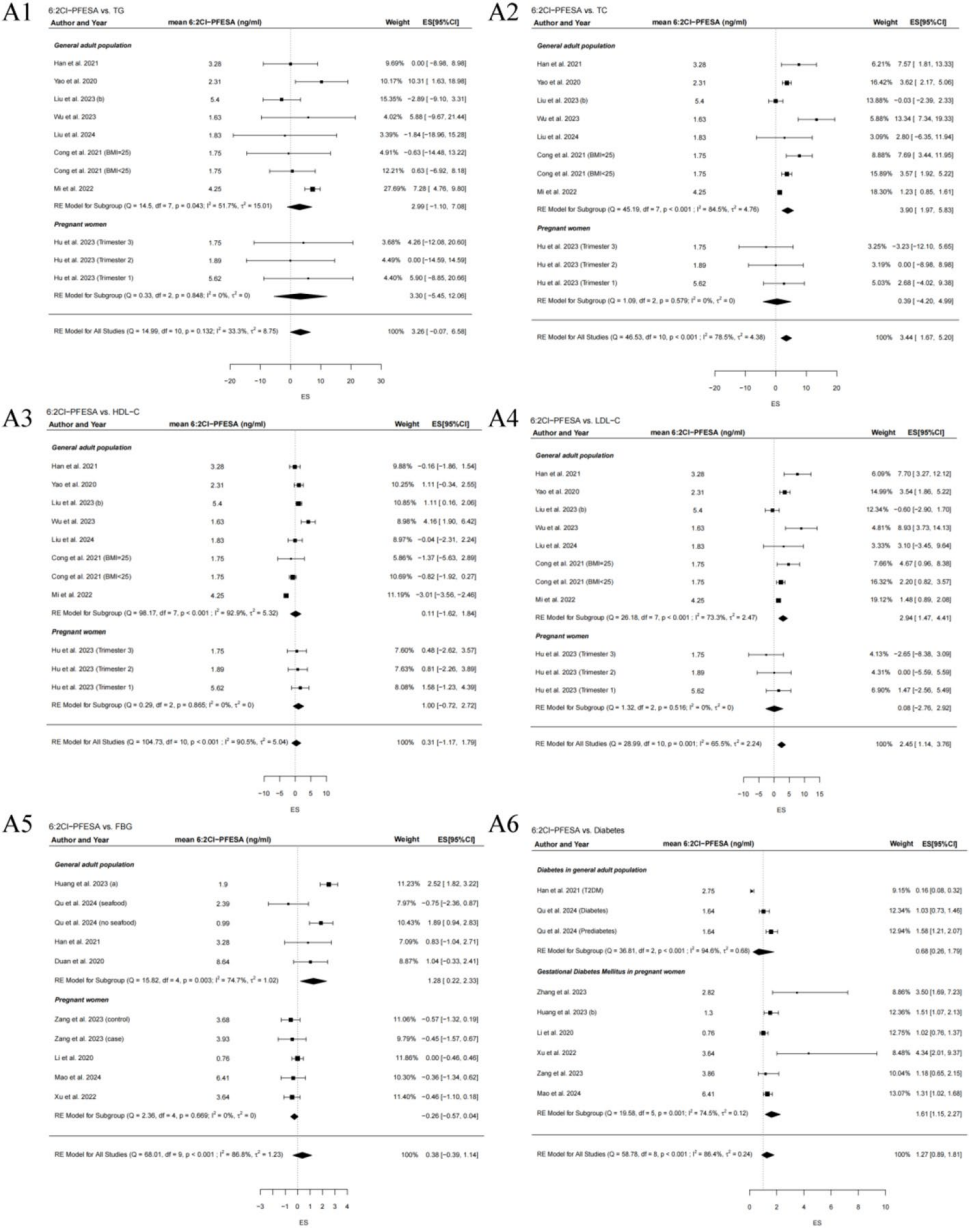
(**A1**-**A5**) Forest plots of pooled estimates of increment (95%CI) of lipids or fasting blood glucose per IQR increase of blood 6:2 Cl-PFESA levels, overall and stratified by different populations (general adult population and pregnant women). (**A6**) Forest plot of pooled OR of diabetes with the highest vs. lowest categories of blood 6:2 Cl-PFESA concentration, overall and stratified by different populations (diabetes in the general adult population, and GDM in pregnant women). Random effects models were used for all analyses. The mean 6:2 Cl-PFESA concentration was represented by the median value extracted from the included studies

The association of 6:2 and 8:2 Cl-PFESA exposure with glucolipid metabolism is summarized in Tables 2– 3 and Fig. 2. Summary of individual datapoints used for main analyses are presented in Table S3-S4. Given the limited evidence currently available for 8:2 Cl-PFESA, the following results focus primarily on the well-characterized 6:2 Cl-PFESA. Overall, significant associations were observed between a per IQR increase in 6:2 Cl-PFESA exposure and higher levels of TC and LDL-C. Per IQR increase of blood 6:2 Cl-PFESA levels was associated with a 3.44 mg/dl change of TC (95%CI: 1.67, 5.20), and a 2.45 mg/dl change of LDL-C (95%CI: 1.14, 3.76). Besides, per IQR increase of 8:2 Cl-PFESA was related to a −0.24 mg/dl change of FBG (95%CI: −0.45, −0.04). Stratified subgroup analyses further indicated significant correlations between 6:2 Cl-PFESA exposure and increased levels of TC (effect size = 3.90, 95%CI: 1.97, 5.83), LDL-C (effect size = 2.94, 95%CI: 1.47, 4.41), and FBG (effect size = 1.28, 95%CI: 0.22, 2.23) in the general adult population, as well as elevated 1 h glucose levels (effect size = 1.99, 95%CI: 0.28, 3.69) and 2 h glucose levels (effect size = 1.89, 95%CI: 0.59, 3.20) in pregnant women. Furthermore, the pooled estimates of subgroup analyses determined a significantly increased risk of GDM among pregnant women exposed to higher 6:2 Cl-PFESA concentration levels (OR = 1.61, 95%CI: 1.15, 2.27). No significant results of 6:2 Cl-PFESA were identified in the overall and subgroup analyses of TG and HDL-C.

**Table 2:** Summary of meta-analyses and subgroup analyses results, which are differences in lipids (mg/dl) per IQR increase of 6:2 Cl-PFESA (95% CI)

Exposure	Outcome	TG		TC		HDL-C		LDL-C	
		No. of studies	Difference (95%CI)	No. of studies	Difference (95%CI)	No. of studies	Difference (95%CI)	No. of studies	Difference (95%CI)
6:2 Cl-PFESA	general adult population	*n* = 7	2.99 (−1.10, 7.08)	*n* = 7	3.90 (1.97, 5.83)*	*n* = 7	0.11 (−1.62, 1.84)	*n* = 7	2.94 (1.47, 4.41)*
	pregnant women	*n* = 1	3.30 (−5.45, 12.06)	*n* = 1	0.39 (−4.20, 4.99)	*n* = 1	1.00 (−0.72, 2.72)	*n* = 1	0.08 (−2.76, 2.92)
	Overall	*N* = 8	3.26 (−0.07, 6.58)	*N* = 8	3.44 (1.67, 5.20)*	*N* = 8	0.31 (−1.17, 1.79)	*N* = 8	2.45 (1.14, 3.76)*

All analyses were conducted using random effects models. The total number of studies is 17. * Effect estimates *p* < 0.05. TG, triacylglycerol

*TC* total cholesterol, *HDL-C* high-density lipoprotein cholesterol, *LDL-C* low-density lipoprotein cholesterol

**Table 3:** Summary of meta-analyses and subgroup analyses results, which are differences in glycemic parameters (mg/dl) per IQR increase of 6:2 and 8:2 Cl-PFESA exposure and pooled OR in diabetes with the highest vs. lowest categories of 6:2 and 8:2 Cl-PFESA concentration (95% CI)

Exposure	Outcome	FBG		1 h glucose level		2 h glucose level		Diabetes	
		No. of studies	Difference (95%CI)	No. of studies	Difference (95%CI)	No. of studies	Difference (95%CI)	No. of studies	OR (95%CI)
6:2 Cl-PFESA	general adult population	*n* = 4	1.28 (0.22, 2.33)*					*n* = 2	0.68 (0.26, 1.79)
	pregnant women	*n* = 4	−0.26 (−0.57, 0.04)	*N* = 4	1.99 (0.28, 3.69)*	*N* = 4	1.89 (0.59, 3.20)* 1.58 (0.23, 2.92)^a^*	*n* = 6	1.61 (1.15, 2.27)*
	Overall	*N* = 8	0.38 (−0.39, 1.14)					*N* = 8	1.27 (0.89, 1.81)
8:2 Cl-PFESA	Overall	*N* = 5	−0.24 (−0.45, −0.04)*	*N* = 4	1.12 (−0.22, 2.46)	*N* = 4	1.19 (−0.04, 2.43) 0.42 (−0.93, 1.76) ^a^	*N* = 5	1.06 (0.91, 1.25)

All analyses were conducted using random effects models. The total number of studies is 17

* Effect estimates *P* < 0.05. *FBG* fasting blood glucose

^a^ Results adjusted for publication bias using the trim-and-fill method

The heterogeneity across studies was noted to be modest-to-high (I^2^ ranging from 65.5 to 78.5%). Univariable meta-regression was performed to explore potential sources of heterogeneity in the significant associations observed for 6:2 Cl-PFESA (Table S8). Sample size was a common moderator across all outcomes. Notably, the explanatory power of other factors varied substantially. ​​For lipids (TC and LDL),​​ heterogeneity was primarily explained by ​​sample size and study design.​​ The lack of a significant moderating effect of population characteristics on lipids is plausibly attributed to the highly skewed distribution of available data, wherein a single study constituted a distinct population group, thereby substantially reducing the statistical power for this comparison. ​​In contrast, for FBG,​​ the distribution of studies across population groups was balanced. The analysis successfully identified ​​participant age, population characteristics, and exposure concentration​​ as significant moderators, explaining a considerable portion of the observed heterogeneity.

### Sensitivity

Sensitivity analyses were conducted by omitting a single article one by one. A detailed analysis for 8:2 Cl-PFESA was not warranted due to data gaps. The overall effect sizes of association between 6:2 Cl-PFESA and TG were significantly changed after excluding articles by Liu [21] and Han [13]. The pooled estimates were 5.97 (95% CI: 3.86, 8.07) and 3.58 (95% CI: 0.02, 7.13), respectively. Surprisingly, the effect size of the association between 6:2 Cl-PFESA and diabetes was significantly changed after excluding the article by Han as well (OR = 1.48, 95%CI: 1.16, 1.89) [13]. This could be attributed to the fact that the covariates of regression models in the article by Han solely comprised age, sex, and BMI. Besides, the restricted number of studies could be a contributing factor. Moreover, the heterogeneity remained high even after the exclusion of the outlier studies by ​​Yao et al.​​ [25] and ​​Wu et al. [20]​​, indicating that these studies were not the primary source of heterogeneity (Table S7). Sensitivity analyses showed that the remaining results were largely unchanged, indicating stable evaluations for most analyses (Figure S1).

### Publication bias

The Egger’s and Begg’s tests detected no significant publication bias for 6:2 Cl-PFESA and 8:2 Cl-PFESA, with the exception of their respective associations with 2 h glucose levels, indicating that most analyses were unaffected (Figure S2, Table S5). Trim-and-fill analysis was therefore conducted for these exposures. The adjusted estimates did not differ materially from the initial results (Table 3), suggesting that the associations remained robust despite detectable bias.

### Meta regression

The Bayesian random-effects meta-regression model was employed to examine dynamic associations of blood 6:2 Cl-PFESA concentrations with (1) TC and LDL-C among the general adult population, and (2) the risk of GDM in pregnant women. Analyses for 8:2 Cl-PFESA were not performed due to insufficient evidence base for robust conclusions. It was regrettable that no significant results were discerned through the meta-regression analyses. The annual change was estimated as −2.53 (−7.51, 2.10) for TC, −2.06 (−4.98, 0.46) for LDL-C, and −0.23 (−1.28, 0.21) for GDM, respectively. The resulting trend plots are shown in Figure S3.

## Discussion

This is the first meta-analysis that synthesizes existing evidence to quantitatively summarize the association of 6:2 and 8:2 Cl-PFESA with glucolipid metabolic alterations. Owing to the limited evidence for 8:2 Cl-PFESA, our analysis focused on 6:2 Cl-PFESA, drawing on studies that involved two distinct populations: the general adult population and pregnant women. Unlike general adults, pregnant women experience an internal state of physiological insulin resistance and hormonal changes. Our subgroup analysis based on population characteristics revealed a distinct divergence in susceptibility: a stronger association was observed between 6:2 Cl-PFESA and lipid metabolism among the general adult population, whereas a more pronounced association with glucose metabolism emerged in pregnant women. This suggests that population-dependent metabolic characteristics may alter the pharmacokinetics of 6:2 Cl-PFESA.

Our analysis indicates that TC and LDL-C may be superior to TG and HDL-C as biomarkers for 6:2 Cl-PFESA-related metabolic effects. Although the individual studies included yielded moderately inconsistent results [19, 21], our pooled estimate revealed statistically significant associations of 6:2 Cl-PFESA with elevated TC (effect size = 3.90) and LDL-C (effect size = 2.94) among adults, which aligns with the positive trends observed in larger studies such as Mi et al. (β for TC and LDL-C per natural log unit increase of 6:2 Cl-PFESA: 0.029 and 0.035) [23]. These effects are comparable to the increases in TC (4.3 mg/dL) and LDL-C (3.1 mg/dL) induced by consuming ​​cheese, a diet high in saturated fat,​​ in a rigorously controlled dietary intervention study, potentially leading to a significant increase in the burden of cardiovascular disease [33]. Findings from animal models, such as those reported by Zhang et al. [34] and Pan et al. [35], demonstrated that exposure to 6:2 Cl-PFESA increased serum LDL-C and hepatic TC levels in mice, further corroborating our findings.

The greater sensitivity of TC and LDL-C likely stems from their lower biological variability and specific metabolic mechanisms. In contrast, TG is more susceptible to acute fluctuations influenced by short-term factors such as immediate dietary intake [36]. This higher variability may account for the discrepancy wherein a significant association between 6:2 Cl-PFESA and TG was observed in animal experiments [34, 35] but not in human epidemiological studies. Results also showed that no significant association was observed between 6:2 Cl-PFESA and HDL-C, suggesting that 6:2 Cl-PFESA likely exerts minimal effect on biological pathways predominantly involving HDL-C.

Our analysis found a significant positive correlation between 6:2 Cl-PFESA and glucose metabolism in pregnant women, including 1 h and 2 h glucose levels, although the limited number of available studies reduces statistical power and warrants cautious interpretation. Furthermore, exposure to 6:2 Cl-PFESA was associated with an increased risk of GDM (OR = 1.61), a finding consistent with that reported by Mao et al. (OR = 1.31) [27]. An OR of 1.61 implies a considerable elevation in risk, comparable to recognized environmental or lifestyle GDM risk factors such as moderate air pollution exposure [37] or high pre-pregnancy BMI [38].

Interestingly, while our analysis revealed a significant positive association between 6:2 Cl-PFESA and FBG, an inverse association was observed for 8:2 Cl-PFESA. This discrepancy may be attributed to the absence of subgroup analyses by population characteristics for the 8:2 Cl-PFESA association. The lack of such stratified assessments stems from the limited number of available studies on 8:2 Cl-PFESA, which precluded a more nuanced examination of potential effect modifiers. This interpretation was further supported by sensitivity analyses (Table S8), which identified population characteristics as a significant source of heterogeneity in the association between 6:2 Cl-PFESA and FBG. Future studies with expanded data on 8:2 Cl-PFESA should aim to clarify the underlying reasons for its opposing association with FBG compared to 6:2 Cl-PFESA, particularly by distinguishing between the influence of structural specificity and potential confounding factors.

In addition, compared to 8:2 Cl-PFESA, exposure to 6:2 Cl-PFESA shows greater effect sizes than PFOS on perturbations of glucolipid metabolism. For example, while previous studies have proved that each IQR increase of PFOS was positively correlated with both TC (effect size = 2.55) and LDL-C (effect size = 1.94) [17], and showed a significant relationship with the incidence of GDM (OR = 1.10) [39], the corresponding effect estimates for 6:2 Cl-PFESA were larger (TC = 3.90, LDL-C = 2.94, GDM = 1.61). The observed effect disparity may be attributed to the enhanced molecular stability based on prolonged half-life (15.3 years) and superior binding affinity toward human transport protein of 6:2 Cl-PFESA relative to PFOS [16, 40].

The significant associations of 6:2 Cl-PFESA with TC, LDL-C, and GDM prompted a Bayesian dose–response meta-regression, but it showed only non-significant negative associations. This could be interpreted as either a genuine absence of a dose–response relationship, or as a reflection of insufficient variability in exposure levels across the studies included in the meta-analysis.

Substantial heterogeneity observed primarily originates from variations in population characteristics, sample size, age, and exposure concentration. Due to differences in excretion pathways, such as menstruation or lactation, the pharmacokinetics of 6:2 Cl-PFESA may vary between sexes. However, a significant effect of sex distribution was observed only in the univariate meta-regression analysis for FBG in our study, with considerable residual heterogeneity remaining (I^2^ = 62.18%). This indicates that FBG may be particularly sensitive to sex-related physiological differences, while also suggesting that factors beyond sex contribute substantially to the observed heterogeneity.

Consequently, our evidence challenges the premise that 6:2 Cl-PFESA is a safe alternative to PFOS, compelling a re-evaluation of the existing risk assessments governing its use. Although epidemiological data remain primarily derived from Chinese populations, environmental monitoring confirms the global dissemination of 6:2 Cl-PFESA [12]. Given its persistent and bioaccumulative characteristics, the observed adverse glucolipid metabolic effects may ​​constitute​​ a worldwide health risk, highlighting the imperative for expanded epidemiological studies to validate these findings across diverse populations.

Although 6:2 Cl-PFESA exhibits metabolic similarities to PFOS, its exact molecular mechanisms have yet to be fully elucidated. Conversely, data regarding 8:2 Cl-PFESA are even more scarce. Consistent with our results, 6:2 Cl-PFESA demonstrated features of greater binding affinity and agonist activity toward PPARs than PFOS, mediating glucolipid metabolism regulation through their associated signaling pathways [6]. Studies in mice and zebrafish have demonstrated that exposure to 6:2 Cl-PFESA induces dyslipidemia, mirroring our findings in human populations. Zhang et al. identified that 6:2 Cl-PFESA exposure in adult male mice disrupted lipid homeostasis via activation of PPARα, ​​by significantly upregulating its downstream target proteins involved in fatty acid oxidation, synthesis, binding, and lipid transport [34]. Pan et al. reported that exposure to 6:2 Cl-PFESA in female mice modulated fatty acid metabolism by activating PPARγ, which subsequently upregulated key genes responsible for de novo fatty acid synthesis and triglyceride production, ultimately promoting lipid synthesis [35]. Research indicated that 6:2 Cl-PFESA modulated key PPAR pathway genes (lpl, slc27a2a, FABP1) by altering gut microbiota (alphaproteobacteria and hyphomicrobium), thereby affecting lipid degradation and synthesis [41]. Apart from this, 6:2 Cl-PFESA exposure was found to elicit oxidative stress responses in zebrafish, thus generating aberrant lipid metabolism [42].

The glucolipid metabolic disruption induced by 6:2 Cl-PFESA likely contributes to GDM pathogenesis through interconnected mechanisms. In contrast, the pathogenic role of 8:2 Cl-PFESA remains less clear due to insufficient evidence. Previous studies have established that GDM correlates with abnormal lipid metabolism in adipose tissue, driven by PPARα in the fatty acid catabolic process and PPARγ in the de novo fatty acid synthesis process [43]. Moreover, elevated oxidative stress in GDM compromises insulin synthesis and signal transduction [44]. Additionally, gestational exposure to 6:2 Cl-PFESA in mice was observed to provoke gut microbiota dysbiosis, exacerbating systemic metabolic disturbances [45]. Furthermore, glucokinase gene mutations have been linked to a heightened risk of GDM among the Chinese population [46]. Experimental studies revealed that 6:2 Cl-PFESA possessed a greater binding affinity to glucokinase than PFOS [7]. This interaction is likely to disrupt glucose homeostasis through impaired glucose perception and insulin secretion in β-cells as well as reduced hepatic glucose utilization, thereby increasing the risk of GDM [47, 48].

Collectively, the mediating role of the PPAR pathway in 6:2 Cl-PFESA’s mechanism of action is well established. In contrast, pathways such as oxidative stress and gut microbiota disruption have only received preliminary support, while the effect on glucokinase remains speculative. Further research is needed to clarify these mechanisms.

Our meta-analysis reveals consistent positive associations between 6:2 PFESA and glucolipid metabolism. However, these associations are derived largely from cross-sectional or case–control designs, which cannot establish temporality and are susceptible to reverse causality. Due to the biological persistence of 6:2 PFESA and its binding to serum proteins, an altered glucolipid metabolic state could affect the distribution of the compound and consequently its measured serum concentration. Though limited, the available prospective evidence does not show a significant association, nor was a monotonic dose–response relationship established. Therefore, while the consistently observed positive association warrants scientific attention, further prospective studies are needed to clarify the nature and direction of this relationship.

## Strengths and limitations

The strengths of our study are as follows. Firstly, it is the first meta-analysis concerning the associations of 6:2 and 8:2 Cl-PFESA with glucolipid metabolism indicators. Secondly, NOS was used to identify the included studies, and a minimum score of 5 was achieved, which reflected their relatively high quality. Furthermore, the current meta-analysis revealed trends with a larger sample size that might not be evident in individual epidemiological studies.

Recognizing the limitations of our study is crucial. First, the limited number of studies and methodological variations, particularly in analytical protocols and limits of detection/quantification (LODs/LOQs) across laboratories, may reduce the precision and comparability of the pooled exposure data. Second, although major confounders were adjusted for in the included studies, residual confounding could remain due to unmeasured or inaccurately assessed factors such as diet, medication use, and insulin resistance. Third, the cross-sectional design of the included studies, combined with exposure assessment based on a single serum measurement of 6:2 Cl-PFESA, limits causal inference. Furthermore, half-life assumptions might not adequately reflect long-term 6:2 Cl-PFESA exposure variability. Finally, since all included studies were conducted in Chinese populations, the generalizability​​ of the findings may be limited.

## Conclusion

In conclusion, our meta-analysis found significant associations of blood 6:2 Cl-PFESA exposure with TC, LDL-C, and GDM. Rigorous longitudinal and multi-omics studies are urgently needed to systematically elucidate its complex toxicological pathways. Given the persistent environmental presence and widespread human detection of 6:2 Cl-PFESA, its association with key metabolic health indicators carries clear public health significance. These findings provide direct scientific evidence to inform the revision and refinement of health risk assessment frameworks for PFAS compounds. Regulatory agencies should fully consider the potential health impacts of 6:2 Cl-PFESA when establishing or updating environmental standards, food safety limits, and occupational exposure guidelines for PFAS.

## Supplementary Information

Supplementary Material 1. Supplementary Appendix 1. PRISMA-P Checklist. Supplementary Appendix 2. PRISMA 2020 Checklist. Supplementary Appendix 3. AMSTAR 2 Checklist. Table S1. Quality of case–control studies included in meta-analysis. Table S2. Quality of cohort studies included in meta-analysis. Table S3. Summary of Individual datapoints used for main analyses of TG, TC, HDL, and LDL. Table S4. Summary of Individual datapoints used for main analyses of FBG, 1 h glucose levels, and 2 h glucose levels. Table S5. Publication bias for 6:2 and 8:2 Cl–PFESA. Table S6. GRADE summary of evidence on the association between 6:2 Cl-PFESA and glucolipid metabolism. Table S7. Sensitivity analysis with the exclusion of outlier studies for 6:2 Cl–PFESA. Table S8. Univariable meta-regression of 6:2 Cl-PFESA with TC, LDL-C, FBG, Diabetes. Figure S1. Leave-one-out analysis for 6:2 Cl–PFESA. Figure S2. Publication bias Begg’s Funnel Plots for 6:2 Cl-PFESA. Figure S3. Bayesian random-effects meta-regression model plots of 6:2 Cl-PFESA with TC, LDL-C, GDM. Figure S4. Log transformation of 6:2 and 8:2 Cl-PFESA exposure and metabolism indexes (Y) for glucose and lipids. Figure S5. Mechanistic links between 6:2 Cl-PFESA exposure and glucolipid metabolism.

## Data Availability

All data generated or analyzed during this study are included in this published article and its supplementary information files.
