# Supplementary material for: Association between 6:2 chlorinated polyfluoroalkyl ether sulfonic acid exposure and glucolipid metabolism in Chinese adults: a meta-analysis
Source: Syst Rev. 2026 Jan 16;15:89. doi: 10.1186/s13643-026-03067-3 (PMC13005563; doi:10.1186/s13643-026-03067-3)
Supplement: Supplementary file 1 — Supplementary Material 1. Supplementary Appendix 1. PRISMA-P Checklist. Supplementary Appendix 2. PRISMA 2020 Checklist. Supplementary Appendix 3. AMSTAR 2 Checklist. Table S1. Quality of case–control studies included in meta-analysis. Table S2. Quality of cohort studies included in meta-analysis. Table S3. Summary of Individual datapoints used for main analyses of TG, TC, HDL, and LDL. Table S4. Summary of Individual datapoints used for main analyses of FBG, 1 h glucose levels, and 2 h glucose levels. Table S5. Publication bias for 6:2 and 8:2 Cl–PFESA. Table S6. GRADE summary of evidence on the association between 6:2 Cl-PFESA and glucolipid metabolism. Table S7. Sensitivity analysis with the exclusion of outlier studies for 6:2 Cl–PFESA. Table S8. Univariable meta-regression of 6:2 Cl-PFESA with TC, LDL-C, FBG, Diabetes. Figure S1. Leave-one-out analysis for 6:2 Cl–PFESA. Figure S2. Publication bias Begg’s Funnel Plots for 6:2 Cl-PFESA. Figure S3. Bayesian random-effects meta-regression model plots of 6:2 Cl-PFESA with TC, LDL-C, GDM. Figure S4. Log transformation of 6:2 and 8:2 Cl-PFESA exposure and metabolism indexes (Y) for glucose and lipids. Figure S5. Mechanistic links between 6:2 Cl-PFESA exposure and glucolipid metabolism. [file 13643_2026_3067_MOESM1_ESM.docx]

**Supplemental Material**

Association between exposure to 6:2 chlorinated polyfluoroalkyl ether sulfonic acid and glucolipid metabolism in Chinese population: a meta-analysis

Qing Chen, Tao Ying, Hua Cai, Hong Liu, Geng-sheng He

**Table of Contents**

**Supplementary Appendix A.** PRISMA-P Checklist

**Supplementary Appendix B.** PRISMA 2020 Checklist

**Supplementary Appendix C.** AMSTAR 2 Checklist

**Table S1.** Quality of case-control studies included in meta-analysis

**Table S2.** Quality of cohort studies included in meta-analysis

**Table S3.** Summary of Individual datapoints used for main analyses of TG, TC, HDL, and LDL

**Table S4.** Summary of Individual datapoints used for main analyses of FBG, 1h glucose levels, and 2h glucose levels

**Figure S1.** Leave-one-out analysis for 6:2 Cl-PFESA

**Figure S2.** Publication bias Begg’s Funnel Plots for 6:2 Cl-PFESA

**Table S5.** Publication bias for 6:2 and 8:2 Cl-PFESA

**Figure S3.** Bayesian random-effects meta-regression model plots of 6:2 Cl-PFESA with TC, LDL-C, GDM

**Figure S4.** Log transformation of 6:2 and 8:2 Cl-PFESA exposure and metabolism indexes (Y) for glucose and lipids

**Table S6.** GRADE summary of evidence on the association between 6:2 Cl-PFESA and glucolipid metabolism

**Table S7.** Sensitivity analysis with the exclusion of outlier studies​ for 6:2 Cl-PFESA

**Table S8.** Univariable meta-regression of 6:2 Cl-PFESA with TC, LDL-C, FBG, Diabetes

**Figure S5.** Mechanistic links between 6:2 Cl-PFESA exposure and glucolipid metabolism

**Supplementary Appendix A.** PRISMA-P Checklist

| **Section/topic** | **#** | **Checklist item** | **Information reported** | | **Line number(s)** |
| --- | --- | --- | --- | --- | --- |
|  |  |  | **Yes** | **No** |  |
| **ADMINISTRATIVE INFORMATION** | | | | | |
| **Title** | | | | | |
| Identification | 1a | Identify the report as a protocol of a systematic review | ✓ |  | 123 |
| Update | 1b | If the protocol is for an update of a previous systematic review, identify as such |  | ✓ |  |
| **Registration** | 2 | If registered, provide the name of the registry (e.g., PROSPERO) and registration number in the Abstract | ✓ |  | 121 |
| **Authors** | | | | | |
| Contact | 3a | Provide name, institutional affiliation, and e-mail address of all protocol authors; provide physical mailing address of corresponding author | ✓ |  | 3-18 |
| Contributions | 3b | Describe contributions of protocol authors and identify the guarantor of the review | ✓ |  | 458-463 |
| **Amendments** | 4 | If the protocol represents an amendment of a previously completed or published protocol, identify as such and list changes; otherwise, state plan for documenting important protocol amendments |  | ✓ |  |
| **Support** | | | | | |
| Sources | 5a | Indicate sources of financial or other support for the review |  | ✓ |  |
| Sponsor | 5b | Provide name for the review funder and/or sponsor | ✓ |  | 451-456 |
| Role of sponsor/funder | 5c | Describe roles of funder(s), sponsor(s), and/or institution(s), if any, in developing the protocol |  | ✓ |  |
| **INTRODUCTION** | | | | | |
| **Rationale** | 6 | Describe the rationale for the review in the context of what is already known | ✓ |  | 51-88 |
| **Objectives** | 7 | Provide an explicit statement of the question(s) the review will address with reference to participants, interventions, comparators, and outcomes (PICO) | ✓ |  | 89-99 |
| **METHODS** | | | | | |
| **Eligibility criteria** | 8 | Specify the study characteristics (e.g., PICO, study design, setting, time frame) and report characteristics (e.g., years considered, language, publication status) to be used as criteria for eligibility for the review | ✓ |  | 127-137 |
| **Information sources** | 9 | Describe all intended information sources (e.g., electronic databases, contact with study authors, trial registers, or other grey literature sources) with planned dates of coverage | ✓ |  | 103 |
| **Search strategy** | 10 | Present draft of search strategy to be used for at least one electronic database, including planned limits, such that it could be repeated | ✓ |  | 105-117 |
| ***STUDY RECORDS*** | | | | | |
| Data management | 11a | Describe the mechanism(s) that will be used to manage records and data throughout the review | ✓ |  | 621 |
| Selection process | 11b | State the process that will be used for selecting studies (e.g., two independent reviewers) through each phase of the review (i.e., screening, eligibility, and inclusion in meta-analysis) | ✓ |  | 118-121 |
| Data collection process | 11c | Describe planned method of extracting data from reports (e.g., piloting forms, done independently, in duplicate), any processes for obtaining and confirming data from investigators | ✓ |  | 139-145 |
| **Data items** | 12 | List and define all variables for which data will be sought (e.g., PICO items, funding sources), any pre-planned data assumptions and simplifications | ✓ |  | 622 |
| **Outcomes and prioritization** | 13 | List and define all outcomes for which data will be sought, including prioritization of main and additional outcomes, with rationale | ✓ |  | 109-113 |
| **Risk of bias in individual studies** | 14 | Describe anticipated methods for assessing risk of bias of individual studies, including whether this will be done at the outcome or study level, or both; state how this information will be used in data synthesis | ✓ |  | 180-199 |
| ***DATA*** | | | | | |
| **Synthesis** | 15a | Describe criteria under which study data will be quantitatively synthesized | ✓ |  | 139-145 |
|  | 15b | If data are appropriate for quantitative synthesis, describe planned summary measures, methods of handling data, and methods of combining data from studies, including any planned exploration of consistency (e.g., *I* ^2^, Kendall’s tau) | ✓ |  | 164-179 |
|  | 15c | Describe any proposed additional analyses (e.g., sensitivity or subgroup analyses, meta-regression) | ✓ |  | 180-199 |
|  | 15d | If quantitative synthesis is not appropriate, describe the type of summary planned |  | ✓ |  |
| **Meta-bias(es)** | 16 | Specify any planned assessment of meta-bias(es) (e.g., publication bias across studies, selective reporting within studies) | ✓ |  | 185-190 |
| **Confidence in cumulative evidence** | 17 | Describe how the strength of the body of evidence will be assessed (e.g., GRADE) | ✓ |  | 159 |

**Supplementary Appendix B.** PRISMA 2020 Checklist

| **Section and Topic** | **Item #** | **Checklist item** | **Location where item is reported** |
| --- | --- | --- | --- |
| **TITLE** | | |  |
| Title | 1 | Identify the report as a systematic review. | Title has been listed in the title of text. |
| **ABSTRACT** | | |  |
| Abstract | 2 | See the PRISMA 2020 for Abstracts checklist. | Structured summary has been briefly outlined in the abstract. |
| **INTRODUCTION** | | |  |
| Rationale | 3 | Describe the rationale for the review in the context of existing knowledge. | Rationale has been shown in the first paragraph of Introduction. |
| Objectives | 4 | Provide an explicit statement of the objective(s) or question(s) the review addresses. | Objectives have been shown in the second paragraph of Introduction. |
| **METHODS** | | |  |
| Eligibility criteria | 5 | Specify the inclusion and exclusion criteria for the review and how studies were grouped for the syntheses. | Eligibility criteria have been described in the inclusion and exclusion criteria (2.1) of method. |
| Information sources | 6 | Specify all databases, registers, websites, organisations, reference lists and other sources searched or consulted to identify studies. Specify the date when each source was last searched or consulted. | Information sources have been listed in the search strategy (2.2) of method. |
| Search strategy | 7 | Present the full search strategies for all databases, registers and websites, including any filters and limits used. | Search has been described in the search strategy (2.2) of method. The exact search strategy is available as Supplementary Material to this paper. |
| Selection process | 8 | Specify the methods used to decide whether a study met the inclusion criteria of the review, including how many reviewers screened each record and each report retrieved, whether they worked independently, and if applicable, details of automation tools used in the process. | Study selection has been shown in figure 1, flow diagram of the study selection process, and search strategy (2.2). |
| Data collection process | 9 | Specify the methods used to collect data from reports, including how many reviewers collected data from each report, whether they worked independently, any processes for obtaining or confirming data from study investigators, and if applicable, details of automation tools used in the process. | Data collection process has been elaborated in the data extraction (2.3) of method. |
| Data items | 10a | List and define all outcomes for which data were sought. Specify whether all results that were compatible with each outcome domain in each study were sought (e.g. for all measures, time points, analyses), and if not, the methods used to decide which results to collect. | Data items have been listed in the Table 1. |
|  | 10b | List and define all other variables for which data were sought (e.g. participant and intervention characteristics, funding sources). Describe any assumptions made about any missing or unclear information. | Data items have been listed in the Table 1. |
| Study risk of bias assessment | 11 | Specify the methods used to assess risk of bias in the included studies, including details of the tool(s) used, how many reviewers assessed each study and whether they worked independently, and if applicable, details of automation tools used in the process. | Methods used for assessing risk of bias were explored in the statistical analysis (2.5) and discussion section of text. |
| Effect measures | 12 | Specify for each outcome the effect measure(s) (e.g. risk ratio, mean difference) used in the synthesis or presentation of results. | Risk ratio, difference in means, etc., have been shown in the result section of meta-analysis. |
| Synthesis methods | 13a | Describe the processes used to decide which studies were eligible for each synthesis (e.g. tabulating the study intervention characteristics and comparing against the planned groups for each synthesis (item #5)). | Methods of data processing and research result’s combination have been described in statistical analysis (2.5). |
|  | 13b | Describe any methods required to prepare the data for presentation or synthesis, such as handling of missing summary statistics, or data conversions. | Methods of data conversions have been described in statistical analysis (2.5). |
|  | 13c | Describe any methods used to tabulate or visually display results of individual studies and syntheses. | Methods of data exhibition have been described in statistical analysis (2.5). |
|  | 13d | Describe any methods used to synthesize results and provide a rationale for the choice(s). If meta-analysis was performed, describe the model(s), method(s) to identify the presence and extent of statistical heterogeneity, and software package(s) used. | Methods of meta-analysis have been described in statistical analysis (2.5). |
|  | 13e | Describe any methods used to explore possible causes of heterogeneity among study results (e.g. subgroup analysis, meta-regression). | Methods used for heterogeneity have been described in statistical analysis (2.5). |
|  | 13f | Describe any sensitivity analyses conducted to assess robustness of the synthesized results. | Methods of sensitivity analyses have been described in statistical analysis (2.5). |
| Reporting bias assessment | 14 | Describe any methods used to assess risk of bias due to missing results in a synthesis (arising from reporting biases). | Methods of publication bias have been described in statistical analysis (2.5). |
| Certainty assessment | 15 | Describe any methods used to assess certainty (or confidence) in the body of evidence for an outcome. | Methods used for certainty have been described in statistical analysis (2.5). |
| **RESULTS** | | |  |
| Study selection | 16a | Describe the results of the search and selection process, from the number of records identified in the search to the number of studies included in the review, ideally using a flow diagram. | Study selection has been described in detail in Figure 1. |
|  | 16b | Cite studies that might appear to meet the inclusion criteria, but which were excluded, and explain why they were excluded. | Study selection has been described in study selection (3.1). |
| Study characteristics | 17 | Cite each included study and present its characteristics. | Study characteristics have been listed in Table 1. |
| Risk of bias in studies | 18 | Present assessments of risk of bias for each included study. | Risk of bias with included studies has been described in detailed in Table S5 and Figure S4-5. |
| Results of individual studies | 19 | For all outcomes, present, for each study: (a) summary statistics for each group (where appropriate) and (b) an effect estimate and its precision (e.g. confidence/credible interval), ideally using structured tables or plots. | Results of individual studies have been shown in Table 2-3, Table S3-4, Figure 2 and Figure S1. |
| Results of syntheses | 20a | For each synthesis, briefly summarise the characteristics and risk of bias among contributing studies. | Results of each synthesis were shown in the result section. |
|  | 20b | Present results of all statistical syntheses conducted. If meta-analysis was done, present for each the summary estimate and its precision (e.g. confidence/credible interval) and measures of statistical heterogeneity. If comparing groups, describe the direction of the effect. | Results of each meta-analysis, including confidence intervals and measures of consistency, were shown in the result section. |
|  | 20c | Present results of all investigations of possible causes of heterogeneity among study results. | Results of all investigations of possible causes of heterogeneity were shown in the result section. |
|  | 20d | Present results of all sensitivity analyses conducted to assess the robustness of the synthesized results. | Results of all sensitivity analyses were shown in the result section. |
| Reporting biases | 21 | Present assessments of risk of bias due to missing results (arising from reporting biases) for each synthesis assessed. | Sensitivity analysis and publication bias were described in the sensitivity analysis and publication bias of result section and Figure S2-5 and Table S5. |
| Certainty of evidence | 22 | Present assessments of certainty (or confidence) in the body of evidence for each outcome assessed. | Results of certainty were shown in the result section. |
| **DISCUSSION** | | |  |
| Discussion | 23a | Provide a general interpretation of the results in the context of other evidence. | Summary of evidence was described in detail in the discussion section. |
|  | 23b | Discuss any limitations of the evidence included in the review. | Limitations of the evidence included in the review were explored in the discussion section. |
|  | 23c | Discuss any limitations of the review processes used. | Limitations of the review processes used were explored in the discussion section. |
|  | 23d | Discuss implications of the results for practice, policy, and future research. | Implications of the results were described in the conclusions section. |
| **OTHER INFORMATION** | | |  |
| Registration and protocol | 24a | Provide registration information for the review, including register name and registration number, or state that the review was not registered. | The review was registered in PROSPERO 2024 CRD42024581843. |
|  | 24b | Indicate where the review protocol can be accessed, or state that a protocol was not prepared. | A protocol can be accessed from: https://www.crd.york.ac.uk/prospero/display_record.php?ID=CRD42024581843. |
|  | 24c | Describe and explain any amendments to information provided at registration or in the protocol. |  |
| Support | 25 | Describe sources of financial or non-financial support for the review, and the role of the funders or sponsors in the review. | This study was funded by the National Key R&D Program of China (2023YFF1104800) and the Key Projects in the Three-year Plan of Shanghai Municipal Public Health System (2023–2025) (GWVI-4). |
| Competing interests | 26 | Declare any competing interests of review authors. | Competing interests of review authors have been described in the declaration of competing interest section. |
| Availability of data, code and other materials | 27 | Report which of the following are publicly available and where they can be found: template data collection forms; data extracted from included studies; data used for all analyses; analytic code; any other materials used in the review. | Availability of data, code and other materials were shown in the supplementary section. |

**Supplementary Appendix C.** AMSTAR 2 Checklist

| **Checklist item** | **Evaluation options** |
| --- | --- |
| 1. Did the research questions and inclusion criteria for the review include the components of PICO? | Yes |
| 2. Did the report of the review contain an explicit statement that the review methods were established prior to the conduct of the review and did the report justify any significant deviations from the protocol? | Yes |
| 3. Did the review authors explain their selection of the study designs for inclusion in the review? | Yes |
| 4. Did the review authors use a comprehensive literature search strategy? | Partial Yes |
| 5. Did the review authors perform study selection in duplicate? | Yes |
| 6. Did the review authors perform data extraction in duplicate? | Yes |
| 7. Did the review authors provide a list of excluded studies and justify the exclusions? | Partial Yes |
| 8. Did the review authors describe the included studies in adequate detail? | Yes |
| 9. Did the review authors use a satisfactory technique for assessing the risk of bias (RoB) in individual studies that were included in the review? | Partial Yes |
| 10. Did the review authors report on the sources of funding for the studies included in the review? | No |
| 11. If meta-analysis was performed did the review authors use appropriate methods for statistical combination of results? | Yes |
| 12. If meta-analysis was performed, did the review authors assess the potential impact of RoB in individual studies on the results of the meta-analysis or other evidence synthesis? | Yes |
| 13. Did the review authors account for RoB in individual studies when interpreting/discussing the results of the review? | Yes |
| 14. Did the review authors provide a satisfactory explanation for, and discussion of, any heterogeneity observed in the results of the review? | Yes |
| 15. If they performed quantitative synthesis did the review authors carry out an adequate investigation of publication bias (small study bias) and discuss its likely impact on the results of the review? | Yes |
| 16. Did the review authors report any potential sources of conflict of interest, including any funding they received for conducting the review? | Yes |

**Table S1.** Quality of case-control studies included in meta-analysis according to the Newcastle-Ottawa Scale

| Author, year | Selection | | | |  | Comparability |  | Exposure | | | Overall score |
| --- | --- | --- | --- | --- | --- | --- | --- | --- | --- | --- | --- |
|  | Definition adequate | Representativeness of the cases | Selection of controls | Definition of controls |  | Comparability of cases and controls |  | Ascertainment of exposure | Same method of ascertainment for cases and controls | Non-Response rate |  |
| Qu et al. 2024 | 1 | 1 | 1 | 1 |  | 2 |  | 1 | 1 | 1 | 9 |
| Liu et al. 2024 | 1 | 0 | 0 | 1 |  | 1 |  | 1 | 1 | 1 | 6 |
| Zhang et al. 2023 | 1 | 0 | 0 | 1 |  | 1 |  | 1 | 1 | 1 | 6 |
| Zang et al. 2023 | 1 | 0 | 0 | 1 |  | 1 |  | 1 | 1 | 0 | 5 |
| Wu et al. 2023 | 1 | 1 | 0 | 1 |  | 2 |  | 1 | 1 | 0 | 7 |
| Liu et al. 2023 (b) | 1 | 1 | 1 | 1 |  | 1 |  | 1 | 1 | 1 | 8 |
| Huang et al. 2023 (a) | 1 | 1 | 1 | 1 |  | 1 |  | 1 | 1 | 1 | 8 |
| Huang et al. 2023 (b) | 1 | 1 | 1 | 1 |  | 1 |  | 1 | 1 | 0 | 7 |
| Xu et al. 2022 | 1 | 1 | 0 | 1 |  | 1 |  | 1 | 1 | 0 | 6 |
| Mi et al. 2022 | 1 | 1 | 1 | 1 |  | 1 |  | 1 | 1 | 1 | 8 |
| Han et al. 2021 | 1 | 1 | 0 | 1 |  | 0 |  | 1 | 1 | 0 | 5 |
| Cong et al. 2021 | 1 | 1 | 1 | 1 |  | 1 |  | 1 | 1 | 1 | 8 |
| Yao et al. 2020 | 1 | 1 | 0 | 1 |  | 2 |  | 1 | 1 | 1 | 6 |
| Duan et al. 2020 | 1 | 1 | 1 | 1 |  | 1 |  | 1 | 1 | 1 | 7 |

**Table S2.** Quality of cohort studies included in meta-analysis according to the Newcastle-Ottawa Scale

| Author, year | Selection | | | |  | Comparability |  | Outcome | | | Overall score |
| --- | --- | --- | --- | --- | --- | --- | --- | --- | --- | --- | --- |
|  | Representativeness of the exposed cohort | Selection of the non-exposed cohort | Ascertainment of exposure | Demonstration that outcome of interest was not present at start of study |  | Comparability of cohorts on the basis of the design or analysis |  | Assessment of outcome | Was follow-up long enough for outcomes to occur | Adequacy of follow up of cohorts |  |
| Mao et al. 2024 | 1 | 1 | 1 | 1 |  | 2 |  | 1 | 1 | 0 | 8 |
| Hu et al. 2023 | 1 | 1 | 1 | 1 |  | 1 |  | 1 | 1 | 0 | 7 |
| Li et al. 2020 | 1 | 1 | 1 | 1 |  | 1 |  | 1 | 1 | 0 | 7 |

**Table S3.** Summary of Individual datapoints used for main analyses of TG, TC, HDL, and LDL

|  |  | TG | | TC | | HDL | | LDL | |
| --- | --- | --- | --- | --- | --- | --- | --- | --- | --- |
| Author, year | Compounds | ES | SE | ES | SE | ES | SE | ES | SE |
| Liu et al. 2024 | 6Cl-PFESA | -1.84 | 8.74 | 2.80 | 4.67 | -0.04 | 1.16 | 3.10 | 3.34 |
| Wu et al. 2023 | 6Cl-PFESA | 5.88 | 7.93 | 13.34 | 3.06 | 4.16 | 1.15 | 8.93 | 2.65 |
| Liu et al. 2023 (b) | 6Cl-PFESA | -2.89 | 3.17 | -0.03 | 1.20 | 1.11 | 0.49 | -0.60 | 1.17 |
| Hu et al. 2023 (Trimester 1) | 6Cl-PFESA | 5.90 | 7.53 | 2.68 | 3.42 | 1.58 | 1.43 | 1.47 | 2.06 |
| Hu et al. 2023 (Trimester 2) | 6Cl-PFESA | 0.00 | 7.45 | 0.00 | 4.58 | 0.81 | 1.57 | 0.00 | 2.85 |
| Hu et al. 2023 (Trimester 3) | 6Cl-PFESA | 4.26 | 8.34 | -3.23 | 4.53 | 0.48 | 1.58 | -2.65 | 2.92 |
| Mi et al. 2022 | 6Cl-PFESA | 7.28 | 1.29 | 1.23 | 0.19 | -3.01 | 0.28 | 1.48 | 0.30 |
| Mi et al. 2022 | 8Cl-PFESA | 1.38 | 1.12 | 0.95 | 0.18 | -1.46 | 0.26 | 1.16 | 0.27 |
| Han et al. 2021 (control) | 6Cl-PFESA | 0.00 | 4.58 | 7.57 | 2.94 | -0.16 | 0.87 | 7.70 | 2.26 |
| Cong et al. 2021 (BMI<25) | 6Cl-PFESA | 0.63 | 3.85 | 3.57 | 0.84 | -0.82 | 0.56 | 2.20 | 0.70 |
| Cong et al. 2021 (BMI<25) | 8Cl-PFESA | 1.36 | 3.46 | 1.18 | 0.75 | -1.18 | 0.60 | 1.18 | 0.60 |
| Cong et al. 2021 (BMI≥25) | 6Cl-PFESA | -0.63 | 7.06 | 7.69 | 2.17 | -1.37 | 2.17 | 4.67 | 1.89 |
| Cong et al. 2021 (BMI≥25) | 8Cl-PFESA | 5.42 | 6.22 | -1.18 | 1.96 | 1.06 | 1.96 | -1.18 | 1.81 |
| Yao et al. 2020 | 6Cl-PFESA | 10.31 | 4.42 | 3.62 | 0.74 | 1.11 | 0.74 | 3.54 | 0.86 |

Notes: Values in the table were differences in lipids (mg/dL) per IQR increase of 6:2 and 8:2 Cl-PFESA exposure and corresponding standard errors. HDL-C, high-density lipoprotein cholesterol; LDL-C, low-density lipoprotein cholesterol; TC, total cholesterol; TG, triacylglycerol; ES, effect size; SE, standard error.

**Table S4.** Summary of Individual datapoints used for main analyses of FBG, 1h glucose levels, and 2h glucose levels

|  |  | FBG | | 1h glucose level | | 2h glucose level | |
| --- | --- | --- | --- | --- | --- | --- | --- |
| Author, year | Compounds | ES | SE | ES | SE | ES | SE |
| Qu et al. 2024 (No seafood) | 6Cl-PFESA | 1.89 | 0.48 |  |  |  |  |
| Qu et al. 2024 (seafood) | 6Cl-PFESA | -0.75 | 0.82 |  |  |  |  |
| Mao et al. 2024 | 6Cl-PFESA | -0.36 | 0.50 | 1.96 | 1.36 | 3.92 | 1.27 |
| Mao et al. 2024 | 8Cl-PFESA | 0.00 | 0.39 | 0.76 | 1.16 | 2.28 | 1.10 |
| Zang et al. 2023 (case) | 6Cl-PFESA | -0.45 | 0.57 | 0.45 | 1.83 | 4.48 | 1.71 |
| Zang et al. 2023 (case) | 8Cl-PFESA | -0.42 | 0.54 | 0.00 | 1.72 | 4.00 | 1.61 |
| Zang et al. 2023 (control) | 6Cl-PFESA | -0.57 | 0.39 | 6.05 | 1.69 | 0.95 | 1.21 |
| Zang et al. 2023 (control) | 8Cl-PFESA | -0.52 | 0.40 | 4.98 | 1.74 | 1.57 | 1.14 |
| Huang et al. 2023 (a) | 6Cl-PFESA | 2.52 | 0.36 |  |  |  |  |
| Xu et al. 2022 | 6Cl-PFESA | -0.46 | 0.33 | 2.01 | 1.03 | 1.19 | 0.93 |
| Xu et al. 2022 | 8Cl-PFESA | -0.26 | 0.13 | 1.40 | 0.93 | 0.33 | 0.82 |
| Han et al. 2021 (control) | 6Cl-PFESA | 0.83 | 0.96 |  |  |  |  |
| Li et al. 2020 | 6Cl-PFESA | 0.00 | 0.24 | 0.46 | 0.86 | 0.92 | 0.67 |
| Li et al. 2020 | 8Cl-PFESA | -0.14 | 0.24 | 0.14 | 0.66 | 0.00 | 0.52 |
| Duan et al. 2020 | 6Cl-PFESA | 1.04 | 0.70 |  |  |  |  |
| Duan et al. 2020 | 8Cl-PFESA | -0.18 | 0.78 |  |  |  |  |

Notes: Values in the table were differences in glycemic parameters (mg/dL) per IQR increase of 6:2 and 8:2 Cl-PFESA exposure and corresponding standard errors. FBG, fasting blood glucose; ES, effect size; SE, standard error.


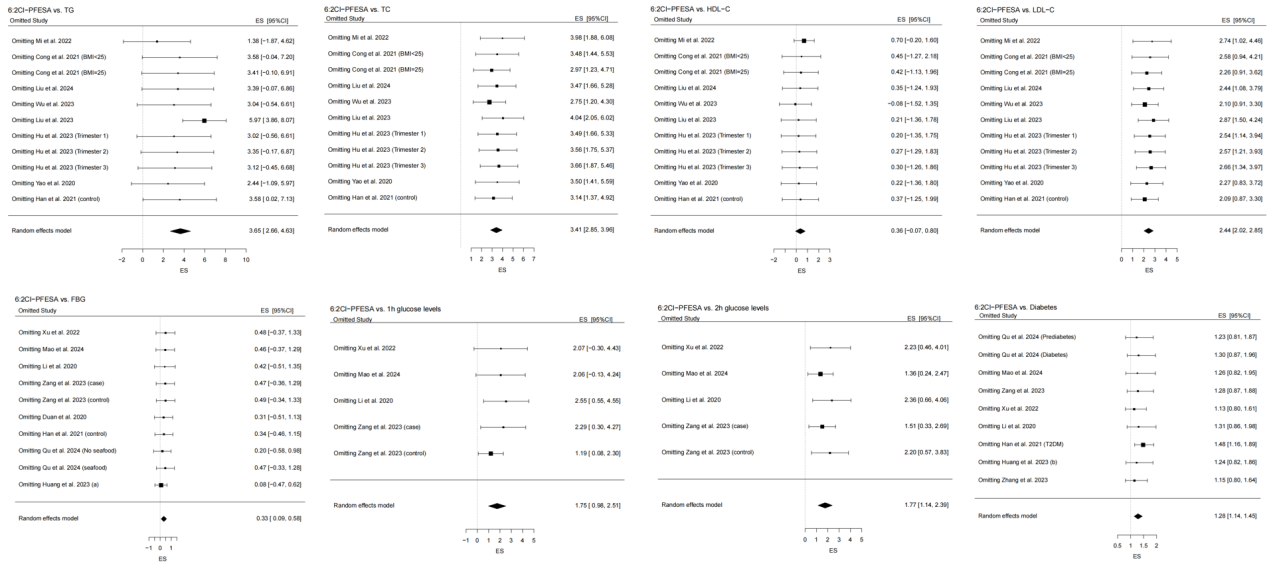


**Figure S1.** Leave-one-out analysis for 6:2 Cl-PFESA

Notes: FBG, fasting blood glucose; HDL-C, high-density lipoprotein cholesterol; LDL-C, low-density lipoprotein cholesterol; TC, total cholesterol; TG, triacylglycerol.


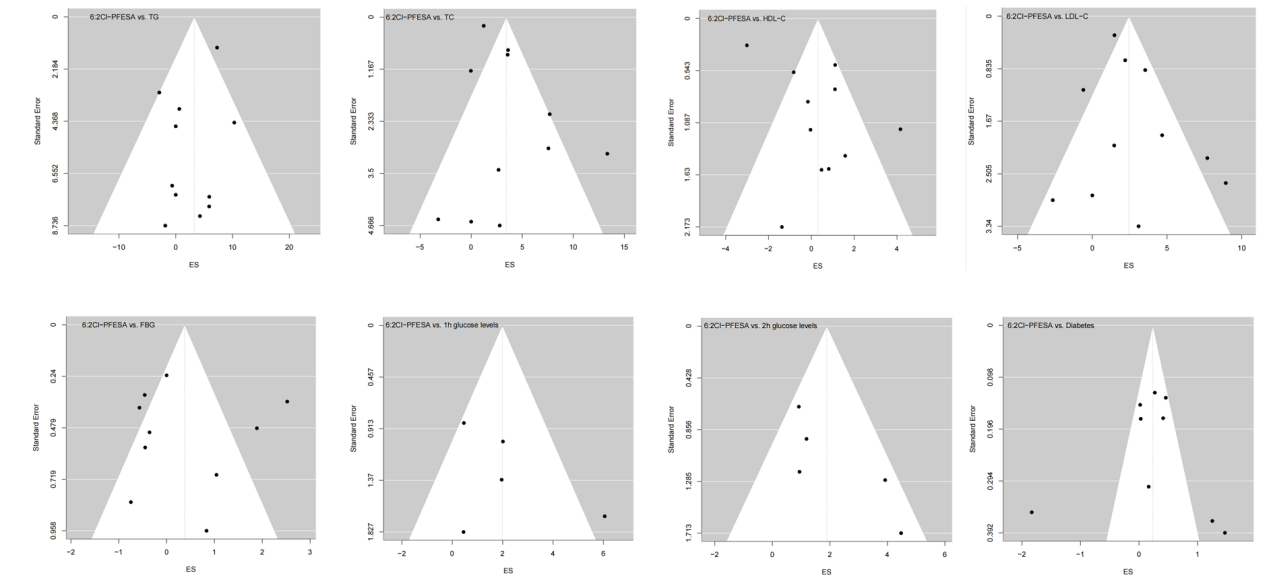


**Figure S2.** Publication bias Begg’s Funnel Plots for 6:2 Cl-PFESA

Notes: FBG, fasting blood glucose; HDL-C, high-density lipoprotein cholesterol; LDL-C, low-density lipoprotein cholesterol; TC, total cholesterol; TG, triacylglycerol.

**Table S5.** Publication bias of the meta-analyses for 6:2 and 8:2 Cl-PFESA

|  | 6Cl-PFESA (*p* value) | | 8Cl-PFESA (*p* value) | |
| --- | --- | --- | --- | --- |
|  | Begg's | Egger's | Begg's | Egger's |
| TG | 0.648 | 0.353 |  |  |
| TC | 0.761 | 0.364 |  |  |
| HDL-C | 0.648 | 0.365 |  |  |
| LDL-C | 0.761 | 0.438 |  |  |
| FBG | 0.291 | 0.949 | 1.000 | 0.972 |
| 1h glucose levels | 0.817 | 0.313 | 0.483 | 0.205 |
| 2h glucose levels | 0.233 | 0.028* | 0.083 | 0.005* |
| Diabetes | 0.612 | 0.789 | 1.000 | 0.885 |

Notes: * Effect estimates *P* < 0.05. FBG, fasting blood glucose; HDL-C, high-density lipoprotein cholesterol; LDL-C, low-density lipoprotein cholesterol; TC, total cholesterol; TG, triacylglycerol.


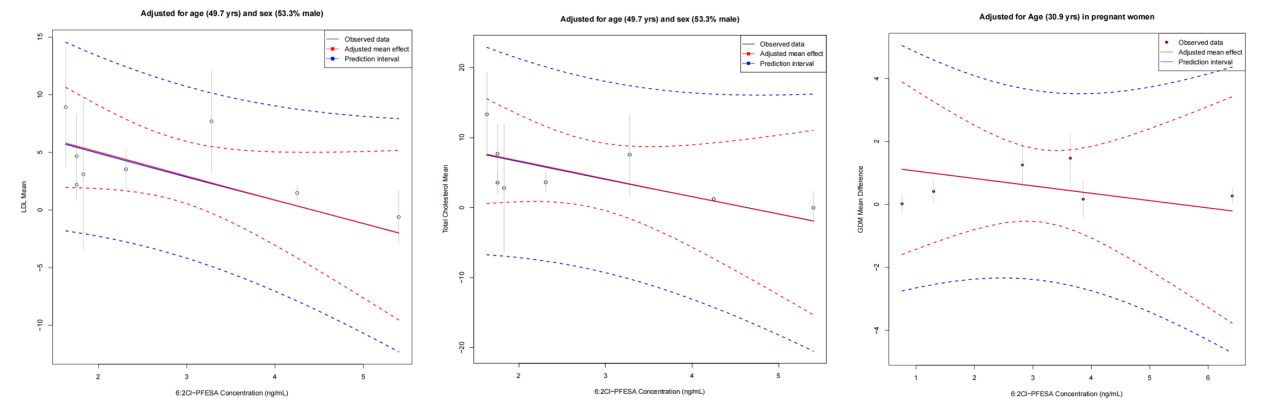


**Figure S3.** Bayesian random-effects meta-regression model plots of 6:2 Cl-PFESA with TC, LDL-C, and GDM

Notes: Plots illustrated the data of 6:2 Cl-PFESA and TC, LDL-C, GDM along with credible intervals for the mean as well as prediction intervals. The mean of 6:2 Cl-PFESA was represented by the median value extracted from the included studies. The model was adjusted for age and sex. FBG, fasting blood glucose; HDL-C, high-density lipoprotein cholesterol; LDL-C, low-density lipoprotein cholesterol; TC, total cholesterol; TG, triacylglycerol; GDM, gestational diabetes mellitus.

**
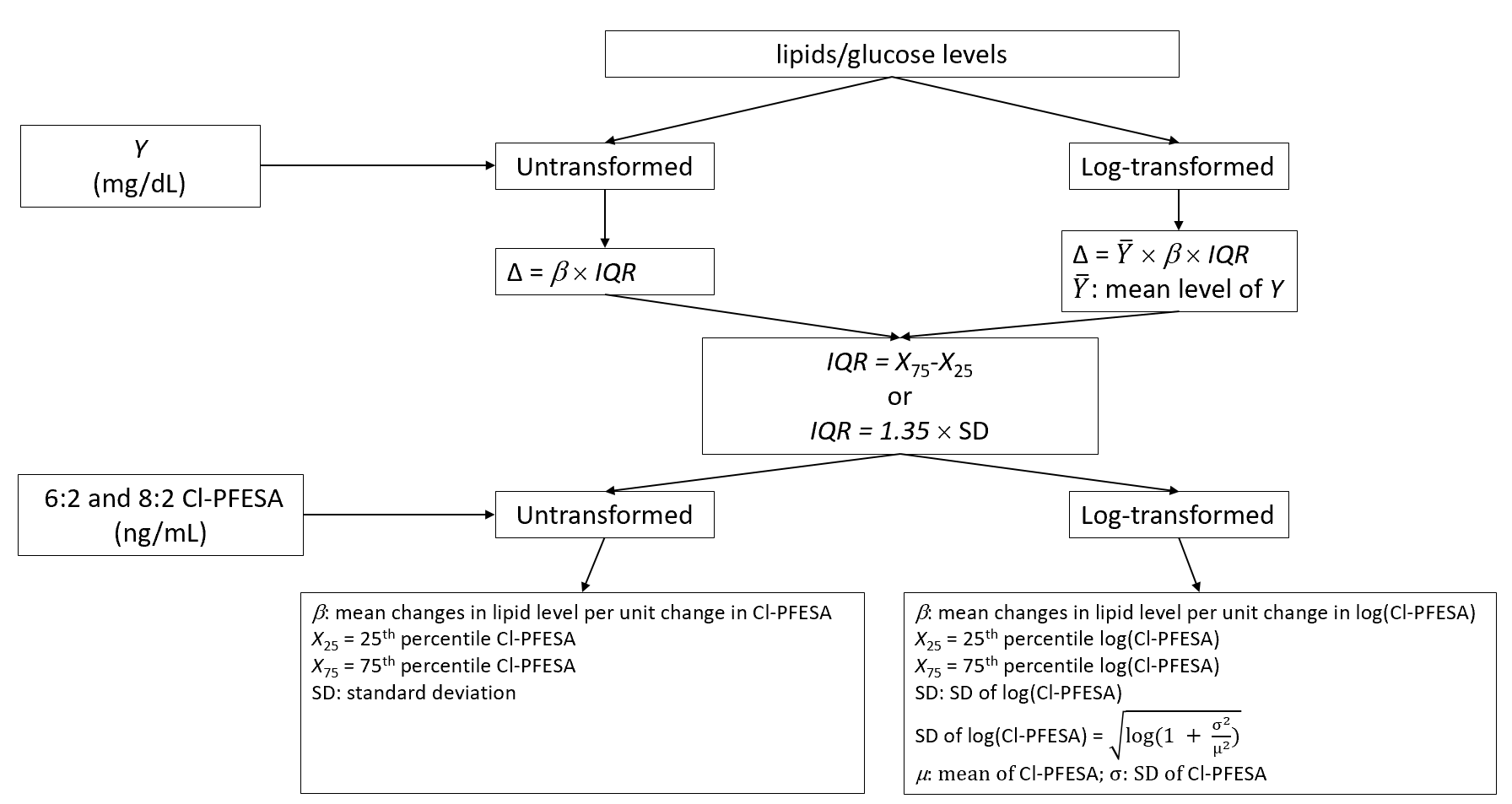
 Figure S4.** Log transformation of 6:2 and 8:2 Cl-PFESA exposure and metabolism indexes (*Y*) for glucose and lipids

Note: The detailed argumentation process is derived from the published literature by Liu et al. Effect in mmol/L lipid unit of TG is multiplied by 88.57 to obtain the effect in mg/dL. Effect in mmol/L lipid unit of TC, HDL-C, LDL-C is multiplied by 38.67 to obtain the effect in mg/dL.

Example: Liu et al., 2024

untransformed 6:2Cl-PFESA (ng/mL): μ=1.829, X_25_= 0.952, X_75_=3.31

untransformed TG (mmol/L): $\bar{Y}$=1.47

ln-transformed TG and ln-transformed 6:2Cl-PFESA: β=-0.013

σ=(3.31-0.952)/1.35=1.747, SD=$\sqrt{LN(1+\frac{\sigma^2}{\mu^2})}$=0.805, IQR=1.087

Δ=1.47*-0.013*1.087*88.57=-1.84

Reference：

Liu B, Zhu L, Wang M, Sun Q. Associations between Per- and Polyfluoroalkyl Substances Exposures and Blood Lipid Levels among Adults-A Meta-Analysis. Environ Health Perspect 2023;131(5):56001 doi: 10.1289/ehp11840.

**Table S6.** GRADE summary of evidence on the association between 6:2 Cl-PFESA and glucolipid metabolism

| **Outcomes** | **Certainty assessment** | | | | | | | **Certainty** |
| --- | --- | --- | --- | --- | --- | --- | --- | --- |
|  | **№ of studies** | **Study design** | **Risk of bias** | **Inconsistency** | **Indirectness** | **Imprecision** | **Other considerations** |  |
| TG | 8 | non-randomised studies | not serious | not serious | not serious | not serious | none | ⨁⨁◯◯ Low |
| TC | 8 | non-randomised studies | not serious | very serious | not serious | not serious | strong association | ⨁◯◯◯ Very low |
| HDL-C | 8 | non-randomised studies | not serious | very serious | not serious | not serious | none | ⨁◯◯◯ Very low |
| LDL-C | 8 | non-randomised studies | not serious | serious | not serious | not serious | strong association | ⨁⨁◯◯ Low |
| FBG | 8 | non-randomised studies | not serious | very serious | not serious | not serious | none | ⨁◯◯◯ Very low |
| Diabetes | 8 | non-randomised studies | not serious | very serious | not serious | not serious | none | ⨁◯◯◯ Very low |

**Table S7.** Sensitivity analysis with the exclusion of outlier studies for 6:2 Cl-PFESA

| Outcomes | Original Qp | Original I^2^ | Adjusted Qp | Adjusted I^2^ | Outlier Study |
| --- | --- | --- | --- | --- | --- |
| TG (general) | 0.043 | 51.7% | 0.042 | 54.1% | Yao et al. 2020 |
| TC (general) | <0.001 | 84.5% | <0.001 | 80.2% | Wu et al. 2023 |
| HDL (general) | <0.001 | 92.9% | <0.001 | 92.1% | Wu et al. 2023 |
| LDL (general) | <0.001 | 73.3% | <0.001 | 68.5% | Wu et al. 2023 |

**Table S8.** Univariable meta-regression of 6:2 Cl-PFESA with TC, LDL-C, FBG, Diabetes

|  | Covariate | Coefficient | CI_LB | CI_UB | *P* Value | I^2^_Remain |
| --- | --- | --- | --- | --- | --- | --- |
| **TC** |  |  |  |  |  |  |
| Age |  | 0.15 | -0.07 | 0.37 | 0.183 | 88.23 |
| Study design | Ref: case-control |  |  |  |  |  |
|  | Cross-sectional | -3.57 | -12.52 | 5.38 | 0.434 | 91.08 |
|  | Longitudinal | -7.41 | -17.78 | 2.96 | 0.162 | 91.08 |
| Population | Ref: general |  |  |  |  |  |
|  | pregnant | -4.16 | -10.59 | 2.27 | 0.205 | 90.13 |
| Sex |  | 0.06 | -0.05 | 0.17 | 0.269 | 87.26 |
| Sample size |  | <0.001 | <0.001 | <0.001 | 0.004 | 76.56* |
| 6:2 Cl-PFESA exposure | Low |  |  |  |  |  |
|  | Medium | -1.76 | -6.43 | 2.90 | 0.459 | 61.59 |
|  | High | -4.77 | -9.18 | -0.37 | 0.034 | 61.59* |
| Number of covariates | Low |  |  |  |  |  |
|  | Medium | -2.33 | -9.48 | 4.82 | 0.523 | 87.21 |
|  | High | -1.31 | -8.71 | 6.08 | 0.728 | 87.21 |
| **LDL** |  |  |  |  |  |  |
| Age |  | 0.11 | -0.04 | 0.27 | 0.142 | 77.95 |
| Study design | Ref: case-control |  |  |  |  |  |
|  | Cross-sectional | -5.10 | -10.82 | 0.61 | 0.080 | 72.78 |
|  | Longitudinal | -7.77 | -14.25 | -1.29 | 0.019 | 72.78* |
| Population | Ref: general |  |  |  |  |  |
|  | pregnant | -3.32 | -7.50 | 0.87 | 0.121 | 80.13 |
| Sex |  | 0.04 | -0.03 | 0.12 | 0.276 | 80.95 |
| Sample size |  | <0.001 | <0.001 | <0.001 | 0.030 | 69.86* |
| 6:2 Cl-PFESA exposure | Low |  |  |  |  |  |
|  | Medium | 0.64 | -2.97 | 4.24 | 0.729 | 55.79 |
|  | High | -2.41 | -5.82 | 1.00 | 0.166 | 55.79 |
| Number of covariates | Low |  |  |  |  |  |
|  | Medium | -3.59 | -7.91 | 0.74 | 0.104 | 73.41 |
|  | High | -2.52 | -6.90 | 1.85 | 0.258 | 73.41 |
| **FBG** |  |  |  |  |  |  |
| Age |  | 0.08 | 0.05 | 0.12 | <0.001 | 44.29* |
| Study design | Ref: case-control |  |  |  |  |  |
|  | cohort | -0.10 | -1.86 | 1.67 | 0.913 | 68.19 |
|  | cross-sectional | 1.49 | -0.13 | 3.11 | 0.071 | 68.19 |
|  | nested case-control | -0.46 | -2.27 | 1.35 | 0.620 | 68.19 |
| Population | Ref: general |  |  |  |  |  |
|  | pregnant | -1.82 | -2.73 | -0.91 | <0.001 | 58.02* |
| Sex |  | 0.03 | 0.01 | 0.05 | <0.001 | 62.18* |
| Sample size |  | <0.001 | <0.001 | <0.001 | 0.599 | 87.13 |
| 6:2 Cl-PFESA exposure | Low |  |  |  |  |  |
|  | Medium | -1.74 | -3.22 | -0.27 | 0.021 | 78.13* |
|  | High | -1.38 | -2.96 | 0.20 | 0.086 | 78.13 |
| Number of covariates | Low |  |  |  |  |  |
|  | Medium | -0.94 | -3.90 | 2.03 | 0.536 | 85.52 |
|  | High | 0.07 | -2.94 | 3.08 | 0.964 | 85.52 |
| **Diabetes** |  |  |  |  |  |  |
| Age |  | -0.03 | -0.08 | 0.02 | 0.210 | 94.16 |
| Study design | Ref: case-control |  |  |  |  |  |
|  | cohort | -0.14 | -2.16 | 1.88 | 0.892 | 96.72 |
|  | cross-sectional | -0.04 | -2.06 | 1.98 | 0.969 | 96.72 |
|  | nested case-control | 0.01 | -2.03 | 2.05 | 0.995 | 96.72 |
| Population | Ref: general |  |  |  |  |  |
|  | pregnant | 0.93 | -0.16 | 2.03 | 0.094 | 93.22 |
| Sex |  | -0.01 | -0.03 | 0.01 | 0.218 | 94.10 |
| Sample size |  | <0.001 | <0.001 | <0.001 | 0.903 | 95.07 |
| 6:2 Cl-PFESA exposure | Low |  |  |  |  |  |
|  | Medium | 0.05 | -1.49 | 1.59 | 0.947 | 96.09 |
|  | High | -0.01 | -1.72 | 1.70 | 0.991 | 96.09 |
| Number of covariates | Low |  |  |  |  |  |
|  | Medium | 0.45 | -1.23 | 2.14 | 0.600 | 95.44 |
|  | High | 0.86 | -0.75 | 2.47 | 0.294 | 95.44 |


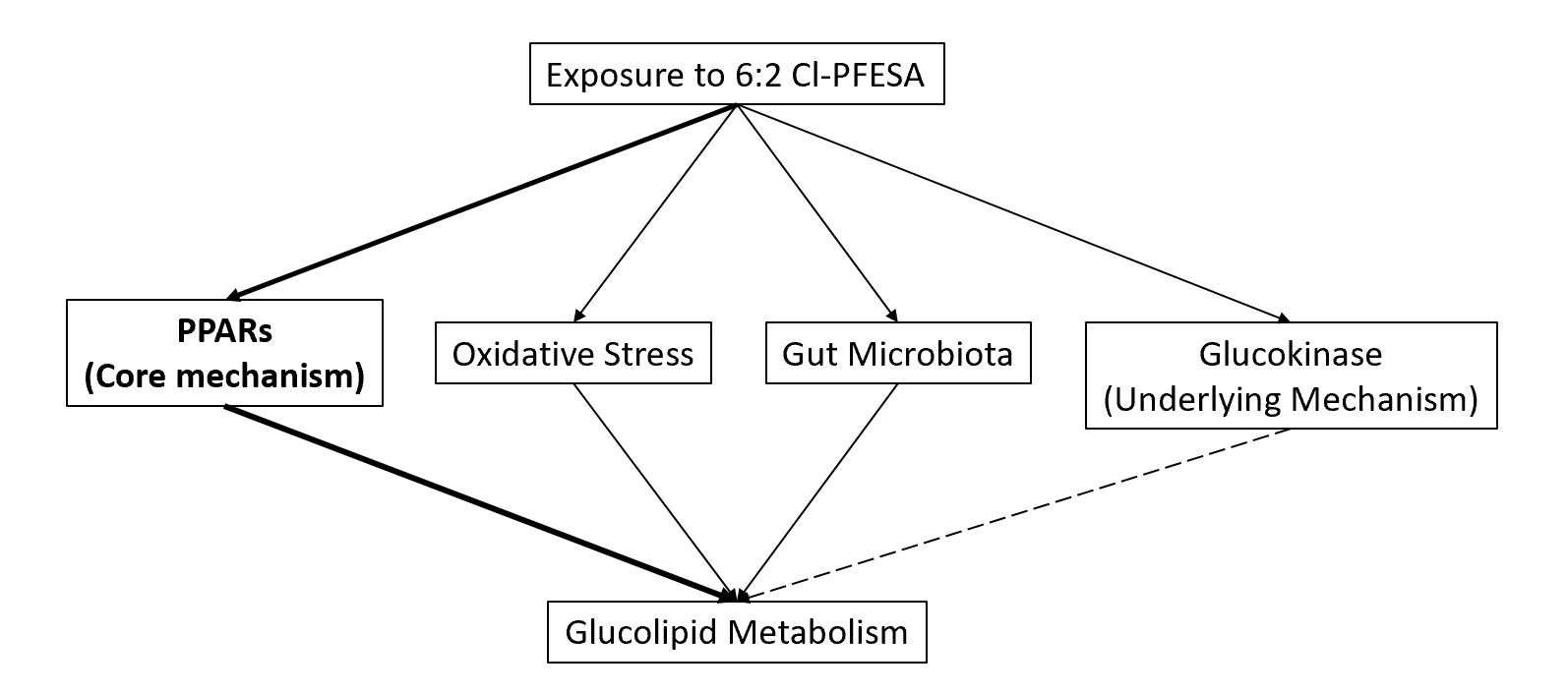


**Figure S5.** Mechanistic links between 6:2 Cl-PFESA exposure and glucolipid metabolism
